# SARS-CoV-2 vaccination may improve anxious, insomnia and depressive symptoms among Chinese population aged 18–75 years during the COVID-19 pandemic

**DOI:** 10.1038/s41598-023-48977-7

**Published:** 2023-12-12

**Authors:** Xiaobo Zhang, Qiang Yue, Mingxia Li, Chaoping Wu, Lu Zhou, Yang Cai, Jian Xu

**Affiliations:** 1 Department of Neurology, Changde Hospital, Xiangya School of Medicine, Central South University (The First People’s Hospital of Changde City), 818 Renmin Road, Changde, 415000 Hunan China; 2 Department of NeurosurgeryChangde Hospital, Xiangya School of Medicine, Central South University (The First People’s Hospital of Changde City), 818 Renmin Road, Changde, 415000 Hunan China; 3 Department of Neurology, Second Xiangya Hospital, Central South University, Changsha, 410011 Hunan China; 4 The Outpatient DepartmentChangde Hospital, Xiangya School of Medicine, Central South University (The First People’s Hospital of Changde City), 818 Renmin Road, Changde, 415000 Hunan China; 5 Department of Infectious Diseases, Changde Hospital, Xiangya School of Medicine, Central South University (The First People’s Hospital of Changde City), 818 Renmin Road, Changde, 415000 Hunan China; 6 Department of Orthopedics, The First Affiliated Hospital, Zhejiang University School of Medicine, Hangzhou, 310003 Zhejiang China

**Keywords:** Psychology, Health care

## Abstract

Previous studies have reported significant decreases in the incidence of mental health problems following SARS-CoV-2 vaccination. However, less relevant studies are published in China. We conducted a cross-sectional study involving Chinese adults aged 18–75 years with no known psychiatric diseases. The study used data from mental health of SARS-CoV-2 vaccinated and unvaccinated participants from May 2020 to July 2021.Three standardized scales, namely, the *Generalized Anxiety Disorder-7* (GAD-7) for anxious symptoms, *Patient Health Questionnaire-9* (PHQ-9) for depressive symptoms and *Athens Insomnia Score-8* (AIS-8) for insomnia symptoms, as well as basic demographic questions were used. The hierarchical regression method was used for multivariate logistic regression analysis to explore the effects of SARS-CoV-2 vaccination on anxious, insomnia, and depressive symptoms. The results confirmed first that vaccinated participants experienced significantly lower anxious, insomnia, and depressive symptoms scores (*P* < 0.001) compared with unvaccinated participants. Second that vaccinated participants had a lower prevalence of anxious, insomnia, and depressive symptoms (*P* < 0.001). Third, after adjusting for potential confounders, we still observed a good correlation between vaccination and a reduced risk of anxious, insomnia, and depressive symptoms. The current study showed that SARS-CoV-2 vaccination may be helpful in improving anxious, insomnia, and depressive symptoms.

## Introduction

The prevalence of COVID-19 has brought unprecedented losses to society and seriously impacted our normal lives. By April 2022, more than 500 million cases had been confirmed worldwide, including more than 6 million deaths^1,2^. In addition, more than 210,000 cases occurred in China, including more than 5000 deaths according to the reported data of Chinese government.The pandemic not only brought the risk of infection and even death to people worldwide but also brought unprecedented psychological distress to humanity^1–3^. Previous studies have confirmed that the incidence rate of psychiatric disorders increased under the influence of the COVID-19 pandemic^2,4–7^. A national cross-sectional survey completed in Sweden showed that anxious symptoms and insomnia symptoms were significantly different, with incidence rates of 24.2% and 38%, respectively^8^. Undoubtedly, the prevalence of anxious symptoms and sleep disorders during the COVID-19 pandemic has been fully proven^9,10^, and people around the world are experiencing more serious mental health problems than before.

To control the spread of the pandemic, restore normal lifestyles as soon as possible, and reduce high mortality and economic losses, vaccination has become the most important and effective measure^11^. A study conducted in Israel showed that two doses of the SARS-CoV-2 vaccine BNT162b2 in more than 6 million people can prevent asymptomatic and symptomatic SARS-CoV-2 infections and reduce the incidence rate, severity, morbidity, and mortality of COVID-19 among all participants^12^. In addition, a study from the United States showed that vaccination with mRNA SARS-CoV-2 vaccine can reduce the possibility of progression to death or mechanical ventilation in hospitalized patients^12^. The results showed that SARS-CoV-2 vaccination could help control the pandemic^13^. Considering that vaccination can lower the risk of infection and mortality, we assume that vaccination against SARS-CoV-2 may reduce the incidence of anxious symptoms, insomnia symptoms, and/or depressive symptoms during the COVID-19 pandemic.

Previous studies have confirmed that the prevalence of anxious symptoms and depressive symptoms among vaccinated people in the United States is significantly lower than that among unvaccinated people^14^. Another study from Bangladesh explored the relationship between vaccination and other mental health problems and found that the prevalence of both anxious symptoms and depressive symptoms reduced after vaccination^9^. As of July, 2021, more than a million doses of SARS-CoV-2 vaccine have been reported according to the CDC data in China. However, to date, limited data have evaluated the effects of SARS-CoV-2 vaccination on mental health problems during COVID-19 in China. To resolve this problem, we investigated whether SARS-CoV-2 vaccination could alleviate the symptoms of anxiety, insomnia, and depression using a web-based tool in China. Our work should provide a better understanding of the effects of SARS-CoV-2 vaccination on mental health problems during the COVID-19 pandemic.

## Methods

### Study participants

A descriptive, cross-sectional survey with non-probabilistic (snow-ball) sampling method was designed to assess the symptoms of anxiety, insomnia, and depression in vaccinated and unvaccinated participants during the COVID-19 pandemic using an anonymous online questionnaire survey platform (“Questionnaire Star”, WeChat app, Tencent Company). The study recruited participants aged 18–75 years by distributing the questionnaire to different WeChat groups and users. We briefly introduced the purpose and significance of the study, as well as the requirements (including age range and whether to participate voluntarily, etc.) before the survey began. Each participant received a survey link that will be useless once the questionnaire was submitted, making this a close-ended survey that did not permitted to be submitted multiple times. Each question was clear and easy to understand in our survey. All participants came from China using WeChat and provided their written informed consent prior to their participation.

**Figure 1:**
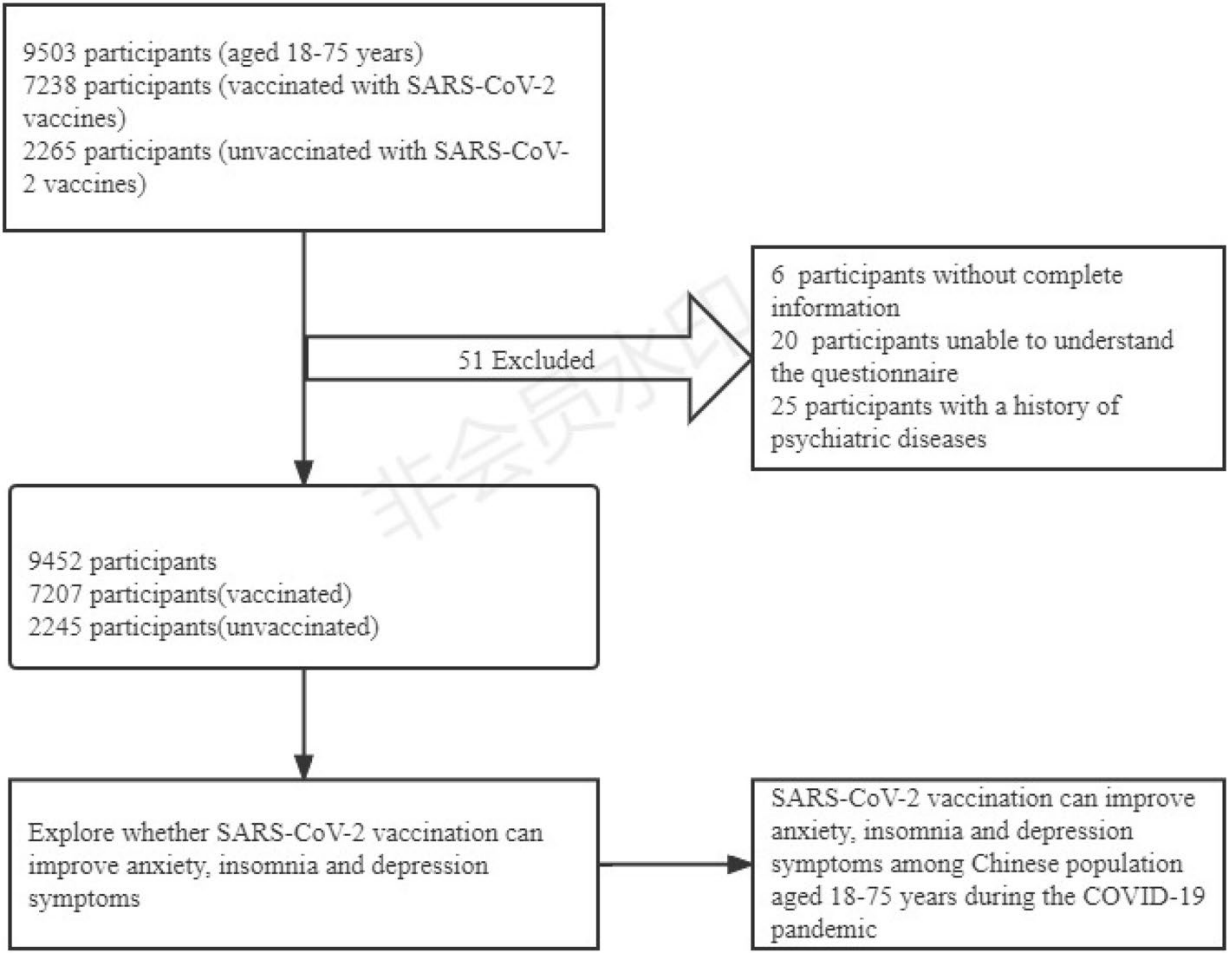
Flowchart of the sample selection.

Participants who met the following conditionswere enrolled: (1) aged 18–75 years, (2) could complete questionnaire survey, (3) WeChat user, and (4) volunteered for the survey. Exclusion criteria included the following: (1) without complete information (2) unable to understand the questionnaire, (3) with a history of psychiatric diseases. The study flowchart is shown in Fig. 1. Ultimately, 9452 questionnaires (response rate, 99.46%) were included in the final analysis. 51 questionnaires were excluded due to missing information or other reasons. This study was approved by the Ethics Committee of Changde Hospital, Xiangya School of Medicine, Central South University (The first people’s hospital of Changde city), Hunan Province, China (No. 2021–040-01). The research was also performed in accordance with the relevant guidelines and regulations.

### Data collection

A standard questionnaire was developed to assess demographic characteristics (age, gender, education level, occupation, marital status, and personal monthly income) and other demographic and socioeconomic indicators, as well as the scores of the GAD-7, AIS-8, and PHQ-9 (explained below). The focus of this research was to understand whether the symptoms of anxiety, insomnia, and depression of all participants can improve after SARS-CoV-2 vaccination. The survey was conducted between May 2020 and July 2021. Ultimately 2245 unvaccinated and 7207 vaccinated individuals were included in this study.

### Survey tools

#### Generalized anxiety disorder-7 (GAD-7)

The GAD-7 is a self-assessment scale for screening and diagnosing anxiety, and it is a 7-item anxious symptoms assessment with scores ranging from 0 to 21^15^. The severity of anxious symptoms can be determined according to the following thresholds:1–4 (minimal symptoms), 5–9 (mild symptoms), 10–14 (moderate symptoms), and ≥ 15 (severe symptoms)^16^. A score of five or above can be used as the critical value for anxiety^17^. This study considered a total score of more than 5 for anxiety. The Cronbach’s alpha for this study was 0.941.

#### Athens Insomnia Scale-8 (AIS-8)

AIS-8 is a self-assessment scale for the screening and diagnosis of insomnia. The scale consisted of eight sub-items^18^. Scores ranged from 0 to 24 based on 0–3 (0 = none, 1 = lowest, 2 = significant, and 3 = severe). A total score of 6 points was considered critical for the diagnosis of insomnia with clinical significance^19^. The Cronbach’s alpha for this study was 0.914.

#### Patient Health Questionnaire-9 (PHQ-9)

We used the Patient Health Questionnaire-9 (PHQ-9) to assess depressive disorders. This instrument includes nine items coded on a scale from 0 to 3 (0 = not at all, 1 = several days, 2 = more than half of the days, and 3 = nearly every day). Total scores ranged from 0 to 27, with a higher score suggesting the presence of a more severe depressive disorder. The depressive symptoms categories were defined as normal (0–4), mild (5–9), moderate (10–14) and severe (≥ 15). A cutoff of ≥ 5 on the PHQ-9 was used as an indicator of clinical depressive disorders^20^. The Cronbach’s alpha for this study was 0.916.

### Statistical analysis

All analyses were performed using SPSS 26.0, Stata/SE 15.0, and the test level α was set to 0.05. Forest plots were drawn using R programming language.

Continuous variables satisfying normality were described as mean ± SD, and an independent sample t-test was used for inter-group comparison; continuous variables that did not meet normality were described by median and quartile (Q1, Q3). Dichotomous or unordered multi-category variables were described by the number of cases (n) and percentage (%), and the groups were compared using the χ^2^ test. Ordinal categorical variables (rank variables: anxious symptoms and depressive symptoms) were described by the number of cases (N) and percentage (%). Ordinal categorical variables (rank variables: anxious symptoms level and depressive symptoms level) were described by the number of cases (N) and percentage (%), and the Wilcoxon rank-sum test was used for comparisons between groups.

Variables with statistical significance (*P* < 0.05) in the above comparison between groups were used as independent variables, and the hierarchical regression method was used for multivariate logistic regression analysis to explore the effect of vaccination on anxious and insomnia symptoms. When anxiety was used as the dependent variable, the second layer was adjusted for age and gender, and the third layer was adjusted for education level, marital status, and personal monthly income. When insomnia was used as the dependent variable, the second layer was adjusted for age and gender; the third layer was adjusted for education level, marital status, personal monthly income, and the fourth level was adjusted for anxious symptoms. When depression was the dependent variable, the second stratum was adjusted for age and gender; the third stratum was adjusted for education level, marital status, and personal monthly income; the fourth stratum was adjusted for anxious symptoms; and the fifth stratum was adjusted for insomnia symptoms.

## Results

### Characteristics of all participants

This study included 9452 participants, of which 2245 (23.8%) were unvaccinated and 7207 (76.2%) were vaccinated. The average age of all respondents was 35.97 ± 11.62 years old, and the male-to-female ratio was 1:1.3. Over two-thirds were from urban areas; about half of the respondents were professional and technical personnel or self-employed/business personnel, 68.5% were married, most participants (85.5%) had a personal monthly income of 10,000 RMB or less, and more than three-fifths had a college/undergraduate education. Among all the respondents, 1676 (17.7%) participants presented with anxious symptoms, 2150 (22.7%) presented with insomnia symptoms, and 2681 (28.4%) had depressive symptoms (Table 1).

**Table 1:** Baseline characteristics of participants.

Characteristics	Total(*n* = 9452)	Vaccination		Statistics	*P-*value
		Before (*n* = 2245)	After (*n* = 7207)		
Gender, *n* (%)				χ = 553.028	< 0.001
Male	4092 (43.3)	1454 (64.8)	2638 (36.6)		
Female	5360 (56.7)	791 (35.2)	4569 (63.4)		
Age (y), mean ± SD	35.97 ± 11.62	30.40 ± 7.92	37.70 ± 12.04	*t* = − 33.224	< 0.001
Residence, *n* (%)				χ = 7.622	0.006
Rural	2697 (28.5)	589 (26.2)	2108 (29.3)		
Urban	6755 (71.5)	1656 (73.8)	5099 (70.7)		
Occupation, *n* (%)				χ = 3246.753	< 0.001
Farming, forestry, animal husbandry, side-line production and fishery practitioners	500 (5.2)	40 (1.8)	460 (6.4)		
Worker	911 (9.6)	246 (10.1)	665 (9.2)		
Professional technicians	3059 (32.4)	326 (14.4)	2733 (37.9)		
Commercial personnel	1991 (21.1)	1339 (59.6)	652 (9.1)		
Civil servant	186 (2.0)	0 (0.00)	186 (2.6)		
Student	743 (7.9)	294 (13.1)	449 (6.2)		
Unemployed	321 (3.4)	0 (0.00)	321 (4.4)		
Other	1741 (18.4)	0 (0.00)	1741 (24.2)		
Marital status, *n* (%)				χ = 402.074	< 0.001
Unmarried	2579 (27.3)	890 (39.6)	1689 (23.4)		
Married	6472 (68.5)	1329 (59.2)	5143 (71.4)		
Divorced	316 (3.3)	0 (0.00)	316 (4.4)		
Widowed	59 (0.6)	0 (0.00)	59 (0.8)		
Other	26 (0.3)	26 (1.2)	0 (0.00)		
Monthly income (CNY), *n* (%)				χ = 590.542	< 0.001
< 5000	4750 (50.2)	697 (31.1)	4053 (56.2)		
5000–10,000	3334 (35.3)	980 (43.6)	2354 (32.7)		
10,000–20,000	832 (8.8)	413 (18.4)	419 (5.8)		
> 20,000	536 (5.7)	155 (6.9)	381 (5.3)		
Education level, *n* (%)				χ = 305.700	< 0.001
High school or below	3108 (32.9)	569 (25.4)	2539 (35.2)		
Junior college or bachelor	6003 (63.5)	1470 (65.5)	4533 (62.9)		
Postgraduate and above	341 (3.6)	206 (9.1)	135 (1.9)		
Depressive symptoms, *n* (%)				χ = 1116.692	< 0.001
No	6771 (71.6)	985 (43.9)	5786 (80.3)		
Yes	2681 (28.4)	1260 (56.1)	1421 (19.7)		
Anxious symptoms, *n* (%)				χ = 1356.131	< 0.001
No	7776 (82.3)	1265 (56.4)	6511 (90.3)		
Yes	1676 (17.7)	980 (43.6)	696 (9.7)		
Insomnia symptoms, *n* (%)				χ = 738.568	< 0.001
No	7302 (77.3)	1375 (56.3)	6362 (83.8)		
Yes	2150 (22.7)	870 (43.7)	1168 (16.2)		

### The symptoms of anxiety, insomnia and depression of participants before and after vaccination

The median of the interquartile range (IQR) of the GAD-7, AIS-8, and PHQ-9 scores of unvaccinated individuals were 3 (0–7), 4 (0–7), and 6 (0, 10), respectively, which were higher than the scores of 0 (0–0), 1 (0–4), and 0 (0, 3) of vaccinated individuals (*P* < 0.001). Moreover, vaccination with the SARS-CoV-2 vaccine significantly reduced the prevalence of anxiety and depression severity ([Z = − 38.05, *P* < 0.001] and [Z = − 35.45, *P* < 0.001]), as detailed in Table 2.

**Table 2:** Anxious, insomnia and depressive symptoms among unvaccinated and vaccinated participants.

Variables	Vaccination		*Z*	*P*
	No	Yes		
GAD-7 (scores)	3 (0, 7)	0 (0, 0)	− 38.66	< 0.001
AIS-8 (scores)	4 (0, 7)	1 (0, 4)	− 25.55	< 0.001
PHQ-9 (scores)	6 (0, 10)	0 (0, 3)	− 32.20	< 0.001
Anxious symptoms degree (N/%)			− 38.05	< 0.001
None	820 (36.5)	5477 (76.0)		
Minimal	445 (19.8)	1034 (14.3)		
Mild	671 (29.9)	573 (8.0)		
Moderate	267 (11.9)	88 (1.2)		
Severe	42 (1.9)	35 (0.5)		
Depressive symptoms degree (N/%)			− 35.45	< 0.001
None	985 (43.9)	5786 (80.3)		
Mild	651 (29.0)	1070 (14.9)		
Moderate	299 (13.3)	205 (2.8)		
Severe	310 (13.8)	146 (2.0)		

### The symptoms of anxiety, insomnia and depression of participants with different characteristics

As shown in Table 3, the prevalence of anxiety among the vaccinated respondents was 9.7%, which was much lower than that among the unvaccinated respondents (43.7%; χ^2^ = 1356.13, *P* < 0.001). The mean age of the participants with anxious symptoms was lower than that of the subjects without anxious symptoms (t = 19.18, *P* < 0.001). In addition, significant differences in the prevalence of anxious symptoms were found among respondents with different gender, occupation, marital status, monthly income, and educational level (*P* < 0.001).

**Table 3:** Anxious symptoms of participants with different characteristics.

Characteristics		Anxious symptoms		*χ*^*2*^*/t*	*P*
		No (N/%)	Yes (N/%)		
Vaccination	No	1265 (56.3)	980 (43.7)	1356.13	< 0.001
	Yes	6511 (90.3)	696 (9.7)		
Age (y)	All	36.9 ± 11.8	31.8 ± 9.4	19.18	< 0.001
Gender	Male	3291 (80.4)	801 (19.6)	16.80	< 0.001
	Female	4485 (83.7)	875 (16.3)		
Residence	Rural	2202 (81.6)	495 (18.4)	1.00	0.317
	Urban	5574 (82.5)	1181 (17.5)		
Occupation	Farming, forestry, animal husbandry, side-line production and fishery practitioners	451 (90.2)	49 (9.8)	512.05	< 0.001
	Worker	792 (86.9)	119 (13.1)		
	Professional technicians	2534 (82.8)	525 (17.2)		
	Commercial personnel	1346 (67.6)	645 (32.4)		
	Civil servant	177 (95.2)	9 (4.8)		
	Student	571 (76.9)	172 (23.1)		
	Unemployed	288 (89.7)	33 (10.3)		
	Other	1617 (92.9)	124 (7.1)		
Marital status	Unmarried	2001 (77.6)	578 (22.4)	87.57	< 0.001
	Married	5423 (83.8)	1049 (16.2)		
	Other	352 (87.8)	49 (12.2)		
Monthly income (CNY)	< 5000	4066 (85.6)	684 (14.4)	115.94	< 0.001
	5000–10,000	2682 (80.4)	652 (19.6)		
	10,000–20,000	591 (71.0)	241 (29.0)		
	> 20,000	437 (81.5)	99 (18.5)		
Education level	High school or technical secondary school or below	2685 (86.4)	423 (13.6)	87.32	< 0.001
	Junior college or bachelor	4857 (80.9)	1146 (19.1)		
	Postgraduate and above	234 (68.6)	107 (31.4)		

As shown in Table 4, the prevalence of insomnia among vaccinated respondents was much lower than that among unvaccinated respondents (16.2% vs. 43.7%) (χ^2^ = 738.56, *P* < 0.001). The prevalence of insomnia symptoms in participants with anxious symptoms was 63.3%, which was significantly higher than that in participants without anxious symptoms (4.3%; χ^2^ = 3961.93, *P* < 0.001). Significant differences in the prevalence of insomnia symptoms were found among respondents with different occupations, marital status, monthly income, and educational level (*P* < 0.001).

**Table 4:** Insomnia of participants with different characteristics.

Characteristics		Insomnia symptoms		*χ*^*2*^*/t*	*P*
		No (N/%)	Yes (N/%)		
Vaccination	No	1263 (56.3)	982 (43.7)	738.56	< 0.001
	Yes	6038 (83.8)	1168 (16.2)		
Age (y)	All	36.8 ± 11.9	33.0 ± 10.1	14.80	< 0.001
Gender	Male	3145 (76.9)	947 (23.1)	0.64	0.422
	Female	4157 (77.6)	1203 (22.4)		
Residence	Rural	2063 (76.5)	634 (23.5)	1.24	0.265
	Urban	5239 (77.6)	1516 (22.4)		
Occupation	Farming, forestry, animal husbandry, side-line production and fishery practitioners	445 (89.0)	55 (11.0)	299.95	< 0.001
	Worker	755 (82.9)	156 (17.1)		
	Professional technicians	2306 (75.4)	753 (24.6)		
	Commercial personnel	1322 (66.4)	669 (33.6)		
	Civil servant	172 (92.5)	14 (7.5)		
	Student	546 (73.5)	197 (26.5)		
	No permanent job or unemployed	267 (83.2)	54 (16.8)		
	Other	1489 (85.5)	252 (14.5)		
Marital status	Unmarried	1903 (73.8)	676 (26.2)	42.81	< 0.001
	Married	5077 (78.4)	1395 (21.6)		
	Other	322 (80.3)	79 (19.7)		
Monthly income (CNY)	< 5000	3803 (80.1)	947 (19.9)	67.25	< 0.001
	5000–10,000	2528 (75.8)	806 (24.2)		
	10,000–20,000	565 (67.9)	267 (32.1)		
	> 20,000	406 (75.7)	130 (24.3)		
Education level	High school or technical secondary school or below	2564 (82.5)	544 (17.5)	94.07	< 0.001
	Junior college or bachelor	4509 (75.1)	1494 (24.9)		
	Postgraduate and above	229 (67.2)	112 (32.8)		
Anxious symptoms	No	6987 (95.7)	315 (4.3)	3961.93	< 0.001
	Yes	789 (36.7)	1361 (63.3)		

In addition, Table 5 shows that vaccinated respondents had a lower prevalence of depressive symptoms symptoms than unvaccinated respondents (19.7% vs. 56.1%) (χ^2^ = 1116.69*, P* < 0.001). The prevalence of depressive symptoms was 93.4% among participants with anxious symptoms and 81.7% among participants with insomnia symptoms, which was significantly higher than those without anxious or insomnia symptoms (*P* < 0.001). In addition, there were significant differences in the prevalence of depressive symptoms among respondents with different occupation, marital status, monthly income, and education level (*P* < 0.001).

**Table 5:** Depressive symptoms of participants with different characteristics.

Characteristics		Depressive symptoms		*χ*^*2*^*/t*	*P*
		No (N/%)	Yes (N/%)		
Vaccination	No	985 (43.9)	1260 (56.1)	1116.69	< 0.001
	Yes	5786 (80.3)	1421 (19.7)		
Age(y)	All	37.5 ± 12.0	32.2 ± 9.6	22.34	< 0.001
Gender	Male	2919 (71.3)	1173 (28.7)	0.32	0.570
	Female	3852 (71.9)	1508 (28.1)		
Residence	Rural	1915 (71.0)	782 (29.0)	0.73	0.390
	Urban	4856 (71.9)	1899 (28.1)		
Occupation	Farming, forestry, animal husbandry, side-line production and fishery practitioners	415 (83.0)	85 (17.0)	456.31	< 0.001
	Worker	714 (78.4)	197 (21.6)		
	Professional technicians	2170 (70.9)	889 (29.1)		
	Commercial personnel	1130 (56.8)	861 (43.2)		
	Civil servant	165 (88.7)	21 (11.3)		
	Student	468 (63.0)	275 (37.0)		
	Unemployed	251 (78.2)	70 (21.8)		
	Other	1458 (71.6)	283 (28.4)		
Marital status	Unmarried	1654 (64.1)	925 (35.9)	115.98	< 0.001
	Married	4815 (74.4)	1657 (25.6)		
	Other	302 (75.3)	99 (24.7)		
Monthly income (CNY)	< 5000	3551 (74.8)	1199 (25.2)	77.79	< 0.001
	5000–10,000	2322 (69.6)	1012 (30.4)		
	10,000–20,000	506 (60.8)	326 (39.2)		
	> 20,000	392 (73.1)	144 (26.9)		
Education level	High school or technical secondary school or below	2458 (79.1)	650 (20.9)	141.96	< 0.001
	Junior college or bachelor	4113 (68.5)	1890 (31.5)		
	Postgraduate and above	200 (58.7)	141 (41.3)		
Anxious symptoms	No	6661 (85.7)	1115 (14.3)	4245.53	< 0.001
	Yes	110 (6.6)	1566 (93.4)		
Insomnia symptoms	No	6377 (87.3)	925 (12.7)	3892.56	< 0.001
	Yes	394 (18.3)	1756 (81.7)		

### Association between vaccination and anxious symptoms

**Figure 2:**
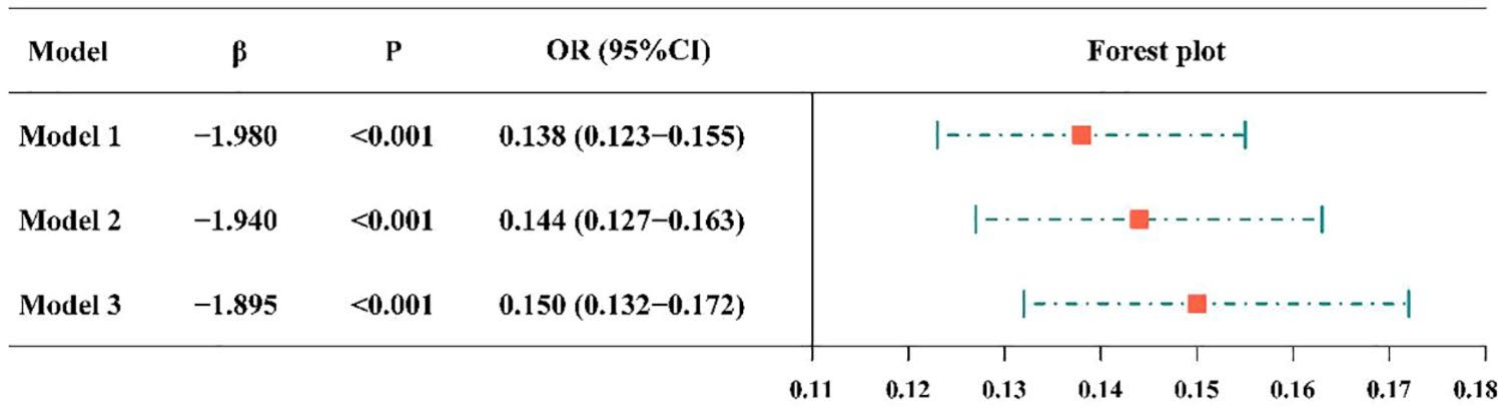
Forest plot showing the association between vaccination and anxious symptoms. CI = confidence interval, OR = odds ratio. The independent variable of Model 1 is vaccination. Model 2 adjusted the age and gender at the base of Model 1, and Model 3 adjusted the education level, occupation, marital status and monthly income at the base of Model 2.

Figure 2 suggests a significant association between vaccination and anxious symptom. Specifically, Model 1 showed that vaccinated respondents had an 86.2% lower risk of anxiety (OR = 0.138, 95% CI 0.123–0.155). After adjusting for age and gender, the risk of anxiety decreased by 0.856 times after vaccination (OR = 0.144, 95% CI 0.127–0.163; Model 2). After further adjustment for known factors such as education level, occupation, marital status, and monthly income based on Model 2, vaccinated respondents had a lower risk of anxiety than unvaccinated respondents (OR = 0.150, 95% CI 0.132–0.172; Model 3).

### Association between vaccination and insomnia symptoms

**Figure 3:**
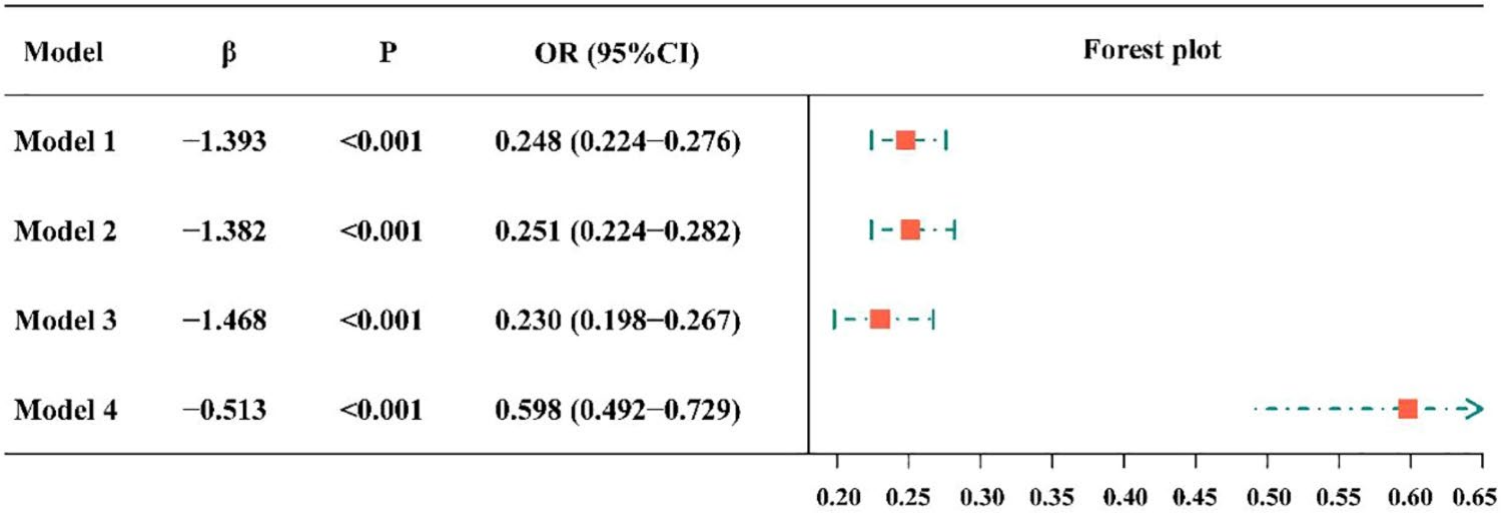
Forest plot showing the association between vaccination and insomnia symptoms. CI = confidence interval, OR = odds ratio. The independent variable of Model 1 is vaccination. Model 2 adjusted the age and gender at the base of Model 1, and Model 3 adjusted education level, occupation, marital status and monthly income at the base of Model 2. Model 4 adjusted anxious symptom at the base of Model 3.

Figure 3 shows a significant association between vaccination and insomnia symptoms. Specifically, Model 1 showed that vaccinated respondents had 75.2% decreased odds of insomnia symptoms (OR = 0.248, 95% CI 0.224–0.276). According to the second model, the risk of insomnia symptoms decreased 0.749 times after vaccination (OR = 0.251, 95% CI 0.224–0.282). Based on Model 3, vaccinated respondents had lower odds of insomnia symptoms than unvaccinated respondents (OR = 0.230, 95% CI 0.198–0.267). After further adjusted for anxious symptoms, Model 4 showed a 40.2% reduction in the odds of insomnia symptoms among vaccinated respondents (OR = 0.598, 95% CI 0.492–0.729).

### Association between vaccination and depressive symptoms

**Figure 4:**
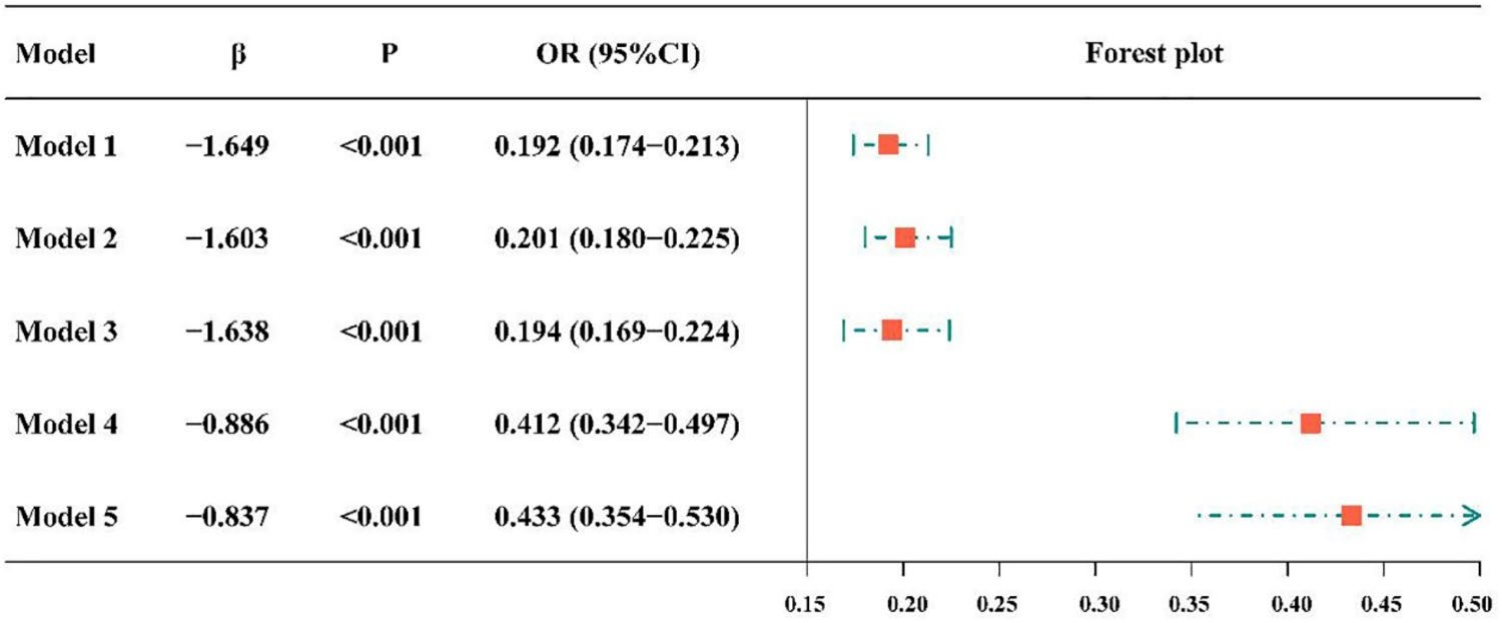
Forest plot showing the association between vaccination and depressive symptoms. CI = confidence interval, OR = odds ratio. The independent variable of Model 1 is vaccination. Model 2 adjusted the age and gender at the base of Model 1, and Model 3 adjusted the education level, occupation, marital status and monthly income at the base of Model 2. Model 4 adjusted anxious symptoms at the base of Model 3. Model 5 adjusted insomnia symptoms at the base of Model 4.

Figure 4 shows a significant association between vaccination and depressive symptoms. Specifically, Model 1 showed that vaccinated respondents had an 80.8% reduced risk of depressive symptoms (OR = 0.192, 95% CI 0.174–0.213). According to Model 2, the risk of depressive symptoms decreased by 0.799 after vaccination (OR = 0.201, 95% CI 0.180–0.225). Based on Model 3, vaccinated respondents had a lower risk of depressive symptoms than unvaccinated respondents (OR = 0.194, 95% CI 0.169–0.224). After adjusting for anxious symptoms, the relationship persisted (OR = 0.412, 95% CI 0.342–0.497), as detailed in Model 4. This association remained significant after adjusting for insomnia symptoms (yes/no) (OR = 0.433, 95% CI 0.354–0.530; Model 5).

## Discussion

In this study, we investigated whether SARS-CoV-2 vaccination has a potentially positive effect on the symptoms of anxiety, insomnia, and depression during the COVID-19 pandemic. To address this issue, we conducted a cross-sectional study that recruited 9425 respondents. This work may be the first largest survey enrolling vaccinated and unvaccinated respondents during the COVID-19 pandemic in China. Our results showed that the scores for anxious, insomnia, and depressive symptoms in vaccinated subjects were significantly lower than those in unvaccinated subjects (all *P* < 0.001). In addition, SARS-CoV-2 vaccination could significantly reduce the prevalence of anxious, insomnia, and depressive symptoms, and mild or greater anxious ([Z = − 38.05, *P* < 0.001] and depressive symptoms [Z = − 35.45, *P* < 0.001]). Furthermore, we observed a good association between SARS-CoV-2 vaccination and a reduced risk of anxious, insomnia, and depressive symptoms. In addition, these associations were independent of potential confounders including age, gender, education level, marital status, personal income, and occupation.

As previously mentioned, our results confirmed that anxious and depressive symptoms scores were significantly reduced after vaccination. These data were consistent with a prior study conducted in Bangladesh^9^, which suggested that vaccinated healthcare workers had significantly lower scores for the symptoms of depression (1.0 [1.0–2.0] vs. 3.0 [2.0–5.0]; *P* < 0.01) and insomnia (3.0 [4.0–7.0] vs. 6.0 [5.0–11.0]; *P* < 0.01). Expecting similar findings, this study further confirmed that the quartile scores of post-traumatic stress disorder and loneliness symptoms were lower in the vaccinated groups with the present work, and that vaccination may effectively alleviate mental health problems. From Tables 3, 4, 5, our findings are also similar to those of another study from the United States, which concluded that the prevalence of anxious and depressive symptoms is higher in unvaccinated participants than in vaccinated participants^14^. A domestic study focusing on 1336 medical students showed that the risk of depression and anxiety symptoms of participants vaccinated twice decreased by 0.6–0.8 times compared with those unvaccinated^21^. The completion of vaccination had a positive influence on mental health outcomes^22^. Despite the limited number of similar studies, the literature showed that the risk of death of vaccinated patients infected with COVID-19 would be significantly reduced^12^. Alternatively, vaccination can reduce constraints during a pandemic and is effective in improving people’s mental health problems. The psychological problems of widowed, divorced, separated, high-income people (monthly income > 10,000 CNY) were alleviated more significantly after SARS-CoV-2 vaccination^23^. However, among people with lower education levels, the relief of anxious and insomnia symptoms caused by vaccination showed more obvious results. Such people lack awareness of the negative effects of the vaccine, and vaccine hesitancy is common in highly educated groups^24^. In addition, vaccination could reduce the probability of insomnia symptoms in our study, but this has not been identified in the studies by Alam et al.^25^ and Wu et al.^21^. The main reasons for this discrepancy may be related to sample size, demographic structure, respondents, and contextual differences.

The model data obtained by hierarchical regression showed that anxious, insomnia, and depressive symptoms risk of vaccinated participants decreased significantly, and vaccination was still statistically correlated with the above psychological problems even after adjusting for several related factors. Previous studies have shown that vaccination can block the transmission of pathogens in addition to protecting oneself^26^. It can not only reduce the economic burden caused by diseases but also form a protective barrier against other infections and further alleviate the pressure on public health and psychological problems^27,28^. In the current situation of the COVID-19 pandemic, vaccination not only reduces the chance of infection and severe illness but also reduces the fear of the COVID-19 pandemic and improves mental health. This is similar to the findings of a previous study in the United States^29^. Although nearly 70 anxiety-related events have been reported in 8624 Janssen COVID-19 vaccine recipients in 2021^30^, which may bring new mental and psychological problems to vaccine recipients at an early stage of vaccination, we believe that any vaccination may lead to anxiety-related events. This observation may simply be a chance occurrence; however, our research has added similar data.

To our knowledge, this maybe the largest sample study in China to explore whether anxious, insomnia, and depressive symptoms can be alleviated after vaccination. Our findings contribute to fill the gap in understanding the impact of SARS-CoV-2 vaccination on mental health status in China. The current study also has some limitations that should be considered in the analysis. First, the cross-sectional study conducted in China could hardly be generalized to other regions and our results can only reflect the situation and influencing factors in the specific period of time (May 2020 to July 2021). Second, the data were collected based on the participants’ self-reports, which may have led to a certain degree of reporting bias. Third, the research population concentrated on respondents aged 18–75 years. Therefore, the research results maybe not extended to other age groups. Fourth, most participants (76.2%) in this study were vaccinated, and only 23.8% were unvaccinated. A large difference in sample size was noted between the two groups, which may reduce the power of the statistical analysis. To compensate for these drawbacks, future research should consider increasing the elderly and minor populations, especially those without vaccination.

In summary, the current study confirmed that SARS-CoV-2 vaccination during the COVID-19 pandemic may be effective in alleviating anxious, insomnia, and depressive symptoms. After adjusting for potential factors, we found a good correlation between vaccination and reduction in anxious, insomnia, and depressive symptoms. In addition, our research may provide favorable conditions for improving vaccination programmes in China and worldwide in the future.

## Data Availability

The raw data supporting the conclusions of this article is available from the corresponding author (Jian Xu) on reasonable request.
